# Identification and validation of telomerase related lncRNAs signature to predict prognosis and tumor immunotherapy response in bladder cancer

**DOI:** 10.1038/s41598-023-49167-1

**Published:** 2023-12-09

**Authors:** Xiaoxu Chen, Zheng Qin, Xiao Zhu, Lili Wang, Changying Li, Haitao Wang

**Affiliations:** 1 Tianjin Institute of Urology, The Second Hospital of Tianjin Medical University, Tianjin, China; 2 Department of Oncology, The Second Hospital of Tianjin Medical University, Tianjin, China

**Keywords:** Bladder cancer, Cancer models

## Abstract

Telomerase allows eukaryotic cells to proliferate indefinitely, an important characteristic of tumor cells. Telomerase-related long no coding RNAs (TERLs) are involved in prognosis and drug sensitivity prediction; however, their association with bladder cancer (BLCA) is still unreported. The objective of this research is to determine a predictive prognostic TERL signature for OS and to provide an efficient treatment option for BLCA. The RNA sequence, clinical information, and mutational data of BLCA patients were acquired from The Cancer Genome Atlas (TCGA) database. With the help of the data from least absolute shrinkage and selection operator (LASSO) regression and Cox regression, a prognostic signature was established including 14 TERLs, which could divide BLCA patients into low-risk (L-R) and high-risk (H-R) cohorts. The time-dependent receiver operating characteristic (ROC) curve demonstrated the greater predictive power of the model. By combing the TERLs-based signature and clinical risk factors (age, sex, grade, and stage), a prognostic nomogram was constructed to forecast the survival rates of patients with BLCA at 1-, 3-, and 5-years, which was well matched by calibration plots C-index and Decision curve analysis (DCA). Furthermore, the L-R cohort showed higher tumor mutation burden (TMB) and lower tumor immune dysfunction and exclusion (TIDE) than the H-R cohort, as well as substantial variability in immune cell infiltration and immune function between the two cohorts was elucidated. As for external validation, LINC01711 and RAP2C-AS1 were identified as poor prognostic factors by survival analysis from the Kaplan–Meier Plotter database, which were validated in BLCA cell lines (EJ, 253J, T24, and 5637) and SV-HUC-1 cells as the control group using qRT-PCR. In addition, interference with the expression of RAP2C-AS1 suppresses the proliferation and migration of BLCA cells, and RAP2C-AS1 could affect the expression of CD274 and CTLA4, which could serve as prognostic markers and characterize the tumor microenvironment in BLCA. Overall, the model based on the 14-TERLs signature can efficiently predict the prognosis and drug treatment response in individuals with bladder cancer.

## Introduction

Bladder cancer (BLCA) is the 10th most frequently diagnosed cancer and the most common malignancy affecting the urinary tract globally^1^. It is known for its high recurrence rate and increased mortality, especially in cases of muscle-invasive bladder cancer (MIBC) classified as stages T2 to T4. The 5-year survival rate for MIBC is less than 50%^2^. Widely used therapies for bladder cancer include radical cystectomy, platinum-based chemotherapy (such as gemcitabine with cisplatin), antibody–drug conjugates (such as RC48), and immune checkpoint inhibitors (ICIs). Despite advances in cancer treatments, objective response rates among patients remain low, and identifying unique biomarkers to predict treatment outcomes remains a challenge^3^. It is particularly crucial to establish a prognostic predictive model to identify the different subtypes of BLCA for precise treatment.

The telomere is a repetitive nucleotide sequence located at the 3' end of a chromosome and consists of TTAGGG. DNA polymerase cannot replicate the DNA ends; therefore, telomeres shorten by about 55 bps after each cell division^4^. This shortening leads to chromosomal instability, which determines the cell division limit before apoptosis. In contrast, telomerase can be activated in specific cell types (intestinal epithelial, germ, endometrium, and tumor cells), causing the replication of telomeric DNA, thus increasing cells' proliferative potential^5^. Telomerase activation is the main mechanism of tumor cell immortalization and is an important step in tumorigenesis^6^. In addition, telomerase function is also associated with epithelial-to-mesenchymal transition (EMT) and cancer stemness, providing tumor cells the ability to metastasize^7^. The catalytic telomerase subunit called telomerase reverse transcriptase (TERT) is critically involved in cancer formation by maintaining telomere homeostasis and cell proliferation potential^8^. Some studies found that TERT expression is associated with immunosuppression and can induce immunosuppressive T cell infiltration, including type 2 T helper, regulatory T (Treg), and myeloid-derived suppressor (MDSCs) cells^9^.

Second-generation sequencing has revealed that 80% of the human genome transcripts are non-coding genes. Long no coding RNAs (lncRNAs) have > 200 nucleotides and modulate gene expression by various mechanisms. Many reports have shown that lncRNAs are linked with progression and BLCA patient's prognosis^10^. Liu et al. reported that lncRNAs participate in different phases of cancer immunity, such as immune activation, immune cell migration, and antigen presentation^11^. However, the relationship between TERLs and BLCA is currently unclear. In this investigation, a survey was conducted to elucidate the role of TERLs in BLCA prognosis and drug sensitivity prediction by constructing a new prognostic signature.

## Materials and methods

### Data acquisition and analysis

The RNA sequence, clinical information, and nucleotide variation data of BLCA individuals were acquired from TCGA database (https://portal.gdc.cancer.gov/ (accessed on 3, May, 2023), comprising 409 tumor and 19 non-tumor samples. Initially, the data was assessed via the Perl language (based on strawberry-perl-5.30; https://www.perl.org) by dividing the transcriptional profile into mRNA and lncRNA matrices. Then, the clinical data was processed using Perl to obtain clinical information and survival data. Tumor mutation burden (TMB) data of BLCA were acquired by processing the nucleotide variation data. Next, Tumor immune dysfunction and exclusion (TIDE) data from the TIDE database (http://tide.dfci.harvard.edu/ (accessed on 3, May, 2023)^12^ were identified using the package "ggpubr" and "limma" for processing. The telomerase gene set was acquired from the Gene set enrichment analysis (GSEA) website (http://www.gsea-msigdb.org/gsea/msigdb/ (accessed on 3, May, 2023)^13^ (Table S1).

### Screening analysis of telomerase-related LncRNAs

First, by using the R package "limma", telomerase genes and the previously obtained mRNA matrix were integrated to obtain a telomerase gene expression matrix. Then, co-expression associations were obtained between the 36 TERs and lncRNA matrix by Pearson correlation analysis (|coefficient| > 0.5, *P* < 0.001). The data of 1053 TERLs and expression were identified, and the co-expression analysis results were depicted in the Sankey diagram prepared by the R package "ggalluvial"^14^.

### Construction and validation of the prognostic model

The R package "limma" was used to integrate the TERLs expression data of BLCA patients with clinical data, resulting in the generation of an expression profile dataset. Subsequently, the R package "caret" was employed to randomly split the data into training and test sets at a 1:1 ratio. Subsequently, univariate and multivariate Cox regression tests, cross-validation, and LASSO algorithm (using the penalty parameter estimated by tenfold cross-validation) were performed to construct a prognostic model for training set (based on R package "glmnet", "survminer", "timeROC", and "survival"), and the model was confirmed by test and total sets. Next, the Akaike Information Criterion (AIC) was used to obtain the expression values and TERLs coefficients, and the risk score for each patient was measured by:

$$riskscore = \sum\limits_{{i = 1}}^{n} {coef\left( i \right)*x(i)}$$

where x(i) = each lncRNA expression, and coef (i) = regression coefficient. The 3 sets (training set, test set, and total set) were then grouped into L-R and H-R cohorts on the basis of median risk score. Next, to validate the influence of the model on the prognosis of BLCA patients, the R package "survival" was applied to conduct a survival analysis on the three sets. Lastly, the prognostic model accuracy was tested by plotting the receiver operating characteristic curve (ROC) by the R packages "survival", "glmnet", "caret", "survminer", "rms", and "timeROC", and calculating the area under the curve (AUC).

### Clinical correlation analysis and construction of the nomogram

To assess whether risk score and clinical factors (grade, sex, stage, and age) affected prognosis in BLCA independently, univariate and multivariate Cox regression were carried out using the R package "survival" and visualized by forest map. Furthermore, with the help of the R package "rms", a nomogram was established to predict the overall survival (OS) of BLCA individuals at 1-, 3-, and 5-years. The calibration curve and C-index assessed the nomogram's predictive power accuracy. Decision curve analysis (DCA) was used to assess the clinical benefit of risk score and clinical factors on patient survival outcomes.

### Principal component and functional enrichment analyses

According to the R package "scatterplot3d", Principal component analysis (PCA) was conducted to classify the expression of TERLs for spatial distribution visualization of L-R and H-R cohorts. GeneOntology (GO) analysis, Kyoto Encyclopedia of Genes and Genomes (KEGG) and GSEA were conducted for the two cohorts on the basis of R packages "enrichplot", "org.Hs.eg.db", "clusterProfiler", "ggplot2", "circlize", "dplyr", "RcolorBrewer", and "ggpubr". Next, gene set variation analysis (GSVA) were conducted based on R package “GSVA”. Lastly, we used the R package “heatmap” to illustrate the enrichment results.

### Tumor immune-related functional analysis

The risk models' tumor immune status was assessed initially via 7 algorithms (CIBERSORT-ABS, TIMER, CIBERSORT, EPIC, and XCELL)^15–21^ to measure immune cells infiltration in the TCGA-BLCA cohort, and the association of prognosis model with immune cell infiltration by R packages "limma", "scales", "ggplot2", "ggtext", "reshape2", "tidyverse", and "ggpubr". Then on the basis of the ESTIMATE algorithm, by the R package "estimate" the abundance of immune and stromal cells in different risk cohorts was assessed. The stromal, immune, and ESTIMATES fractions (stromal fraction + immune fraction) were analyzed^22^. Followed by single-sample GSEA (ssGSEA) scoring of immune functions in the TCGA-BLCA dataset were performed by R packages "GSVA", "GSEABase", and "limma"^23^, the input file "immune. gmt" was downloaded from the GSEA database. Finally, with the R package "ggpubr" potential ICIs responses between the risk cohorts were assessed.

### Tumor immune dysfunction and exclusion score and tumor mutation burden

The Perl language was used to extract BLCA mutation data from TCGA. The R package "maftools" was applied to identify the TMB and survival data for both risk cohorts^24^. The TIDE score files were acquired from the TIDE website and identified via the R package "ggpubr". Next, the data of pan-cancer cancer stem cell (CSC) scores were acquired from the UCSC xena (http://xena.ucsc.edu/ (accessed on 3, May, 2023) database, and for analysis, R packages "ggplot2", "ggpubr", "limma", "ggExtra" were used.

### IC50 prediction of the different drug therapy

Using the R package "pRRophetic", the gene expression levels were utilized to determine the half-maximal inhibitory concentration (IC50) of the targeted drug as a reflection of therapeutic sensitivity ^25^ based on Genomics of Drug Sensitivity in Cancer (GDSC) (https://www.cancerrxgene.org/ (accessed on 3, May, 2023).

### Cell line culture

Normal human bladder epithelial immortalized cells (SV-HUC-1) and bladder cancer cell lines (EJ, 253J, T24, 5637) were obtained from the Institute of Urology, Second Hospital of Tianjin Medical University. SV-HUC-1 cells were cultured in F-12K medium (Meilunbio, Dalian, China). EJ, 253J, and 5637 cells were cultured in RPMI-1640 medium (Transgene, Beijing, China), and T24 cells were cultured in McCoy’s 5A medium (VivaCell, Shanghai, China). All the media were supplemented with 10% fetal bovine serum (Transgene, Beijing, China) and 1% streptomycin/penicillin (Basalmedia, Shanghai, China), and all the cells were incubated in a humidified atmosphere with 5% CO_2_ at 37 °C.

### Quantitative real-time polymerase chain reaction (qRT-PCR)

The cell lines (SV-HUC-1, EJ, 253J, T24, and 5637) were used to extract total RNA using TRIzol Reagent (Transgene, Beijing, China) following the manufacturer's instructions. The HiFiScript RT cDNA Synthesis Kit (CWBIO, Beijing, China) was used to generate cDNA according to the manufacturer's protocol. TOROGreen qPCR Master Mix (TOROIVD, Shanghai, China) was used for qRT-PCR. The 2-ΔΔ method was used to determine the relative RNA expression, with GAPDH as the internal control. The following primer sequences were used: RAP2C-AS1-F: 5ʹ-ACTTAGCCGTGCCTGACAAA-3ʹ, RAP2C-AS1-R: GCTCCAAAAAGGCACCCTTG; LINC01711-F: 5ʹ-GGTCTGGAGCCGTTTCTCTC-3ʹ, LINC01711-R: 5ʹ-GGAGGAGAGGGGTTCTCCAT-3ʹ; GAPDH-F: 5ʹ-GGGGAGCCAAAAGGGTCATCATCT-3ʹ, GAPDH-R: 5ʹ-GACGCCTGCTTCACCACCTTCTTG-3ʹ. CD274-F: 5ʹ-CCATACAGCTGAATTGGTCATCCC-3ʹ, CD274-R: 5ʹ-GAATGTCAGTGCTACACCAAGGC-3ʹ; CTLA4-F: 5ʹ-TTGGATTTCAGCGGCACAAGGC-3ʹ, CTLA-4-R: 5ʹ-TGCTGGCCAGTACCACAGCAGG-3ʹ.

### RNA interference assay

Small interfering RNA (siRNA) probe against RAP2C-AS1 was developed and synthesized by GenePharma (Suzhou, China). The sequences were: RAP2C-AS1-Si-1: sense: 5ʹ-GCAUAUAUCAGUGGAUCUUTT-3ʹ, antisense: 5ʹ-AAGAUCCACUGAUAUAUGCTT-3ʹ; RAP2C-AS1-Si-2: sense: 5ʹ-GCAUACACACAGAUCAAUATT-3ʹ, antisense: 5ʹ-UAUUGAUCUGUGUGUAUGCTT-3ʹ. All transfections were carried out with RFect Transfection Reagent (Baidai, Changzhou, China). Cells were collected 48h after transfection and transfection efficiency was verified using qRT-PCR.

### CCK8 and Colony formation

BLCA cells of logarithmic growth phase were taken, normal control (NC) and treated cells were digested and resuspended to single cell suspension and counted. We added equal amounts of cell suspension (1000 cells/well) to 96-well plates with 3 sub-wells per group and placed in a cell incubator (37°C, 5% CO_2_). The plates were incubated for 24 h, 48 h, 72 h and 96 h in a cell-incubator. Add 200 μl of assay solution (180 μl complete medium + 20 μl CCK8 reagent) to each hole and put the plates back into the incubator for another 2 h or so. After the incubation, absorption at 450 nm was measured at different times using an enzyme tagger. Colony formation was detected after 7–10 days of incubation of 800 cells/well in 6-well plates. The cells were fixed with methanol and stained with a crystal violet solution. Clones containing at least 50 cells were counted using ImageJ.

### Transwell assay

After centrifuging the cell suspension obtained by digestion, the upper medium was removed, and the cells were resuspended in a serum-free medium; 200 µl (approxi-mately 1 × 10^5^ cells) were inoculated into the upper chamber, and then 20% FBS-containing culture (500 µl) was added to the lower chamber. After culturing 24 h of culture, the number of migrating cells in the selected domain was counted using ImageJ.

### Wound healing assay

Cells were seeded in 6-well plates (4 × 10^5^ cells/well). When the cell confluence reached 100%, the fused cells were scratched with a 200 μl pipette tip, the detached cells were washed off with PBS, and finally, pictures of the scratches were taken at 0 h and 24 h with an inverted microscope (Canon EOS 800D, Tokyo, Japan). ImageJ was used to count the migration distances in the selected fields.

### Statistical analysis

Statistical measurements were conducted via Perl language and R (version 4.2.1, http://www.Rproject.org) software. All experiments were repeated three times independently, and the data were processed using GraphPad Prism 8 (GraphPad Software, Inc). The results were presented in the form of mean ± standard deviation, student t-test (for independent samples), and one-way ANOVA (for analysis of variance) was used for comparison between groups.* P* < 0.05 was considered to indicate a statistically significant difference.

### Ethics approval and consent to participate

This study was obtained from the public databases TCGA, TIDE and GSEA, and all methods followed the relevant guidelines and regulations.

## Results

### Identification of telomerase related lncRNAs signature

**Figure 1:**
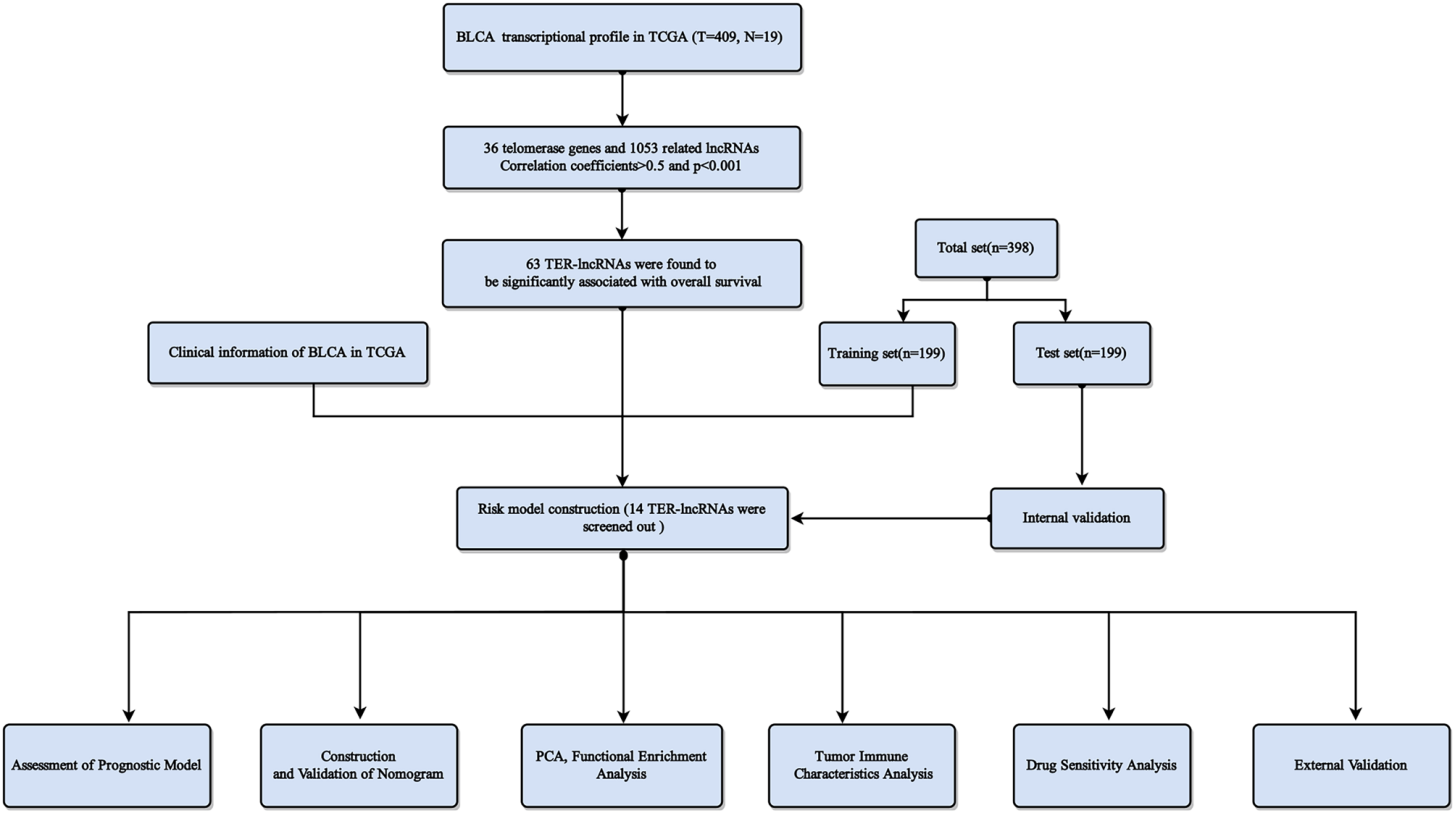
Research process.

**Figure 2:**
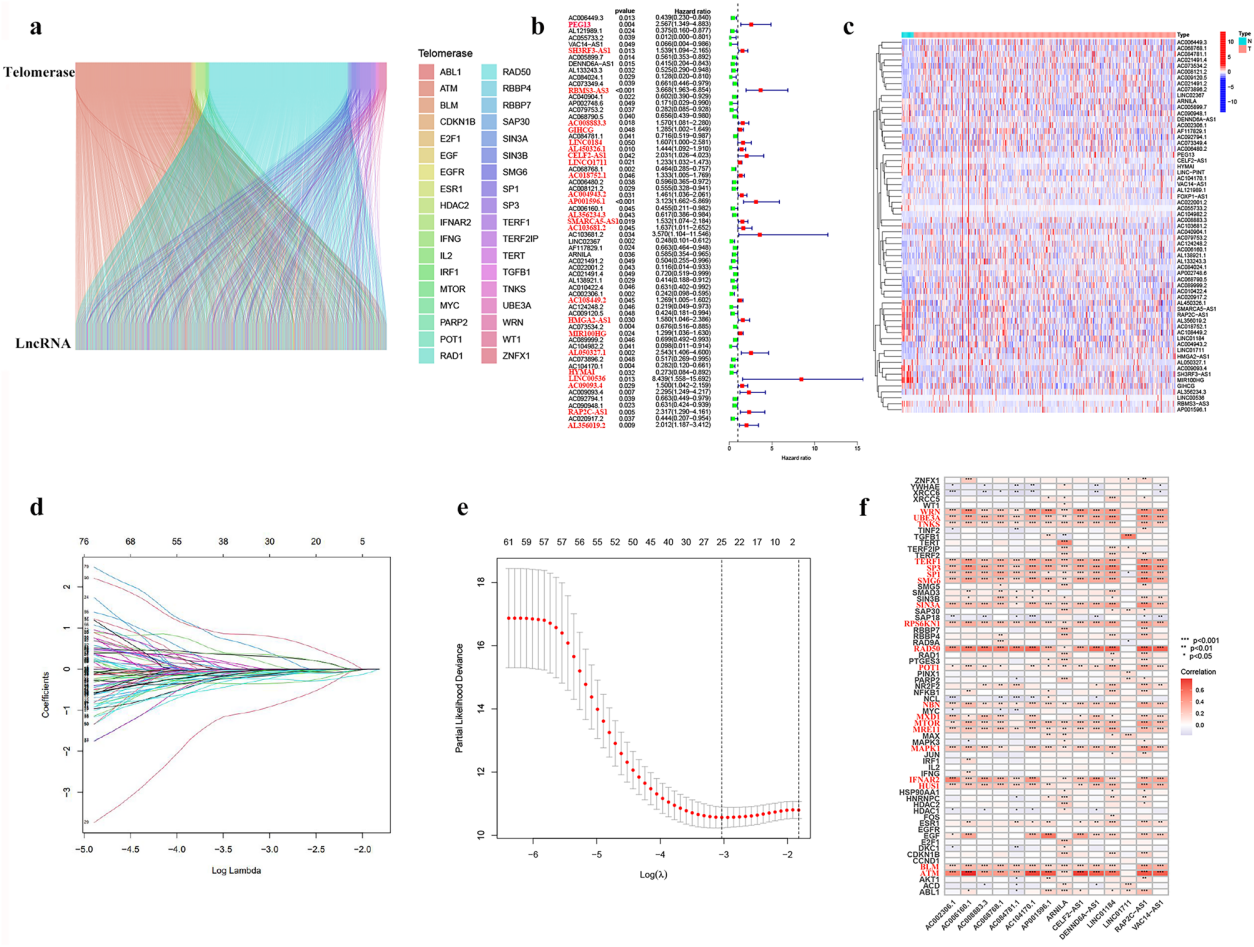
Determination of TERLs and BLCA prognostic value. (**a**) 1053 lncRNAs co-expressed with 36 telomerase-associated genes. (**b**) 63 TERLs associated with prognosis in the training set. (**c**) The expression profiles of these TERLs in tumor and non-tumor samples. (**d**) The LASSO coefficient data of TERLs. (**e**) Cross-validation for LASSO model variable selection. (**f**) Correlation of TERLs with telomerase-related genes.

Figure 1 depicts the research process. 1053 lncRNAs co-expressed with 36 telomerase genes (ABL1, ATM, BLM, CDKN1B, E2F1, EGF, EGFR, ESR1, HDAC2, IFNAR2, IFNG, IL2, IRF1, MTOR, MYC, PARP2, POT1, RAD1, RAD50, RBBP4, RBBP7, SAP30, SIN3A, SIN3B, SMG6, SP1, SP3, TERF1, TERF2IP, TERT, TGFB1, TNKS, UBE3A, WRN, WT1, ZNFX1) in BLCA (|Pearson R| > 0.5 and *P* < 0.001) were identified. The Sankey diagram represents the data obtained from the co-expression analysis (Fig. 2a). We performed univariate Cox regression analysis on 63 TERLs associated with prognosis in the training set. Among them, 24 TERLs (LINC01711, AC108449.2, GIHCG, MIR100HG, AC018752.1, AL450326.1, AC004943.2, LINC00536, AL356234.3, SH3RF3-AS1, AC008883.3, HMGA2-AS1, LINC01184, SMARCA5-AS1, AL356019.2, CELF2-AS1, AC009093.4, RAP2C-AS1, AL050327.1, PEG13, AP001596.1, AC103681.2, RBMS3-AS3, HYMAI) were identified as risk factors with a hazard ratio (HR) greater than 1, while 39 TERLs were identified as protective factors with an HR less than 1. To visualize these findings, a forest plot was generated. (Fig. 2b). The expression profiles of these TERLs in tumor and non-tumor samples were shown in Fig. 2c. Furthermore, by the LASSO algorithm based on the training set, 25 lncRNAs were obtained, 14 of which were further performed for the multivariate Cox proportional to construct a risk model (Fig. 2d,e), Finally, the relationship between 36 telomerase-related genes and 14 lncRNAs of the prognostic model were shown in Fig. 2f.

### Clinical characteristics and survival analysis

**Figure 3:**
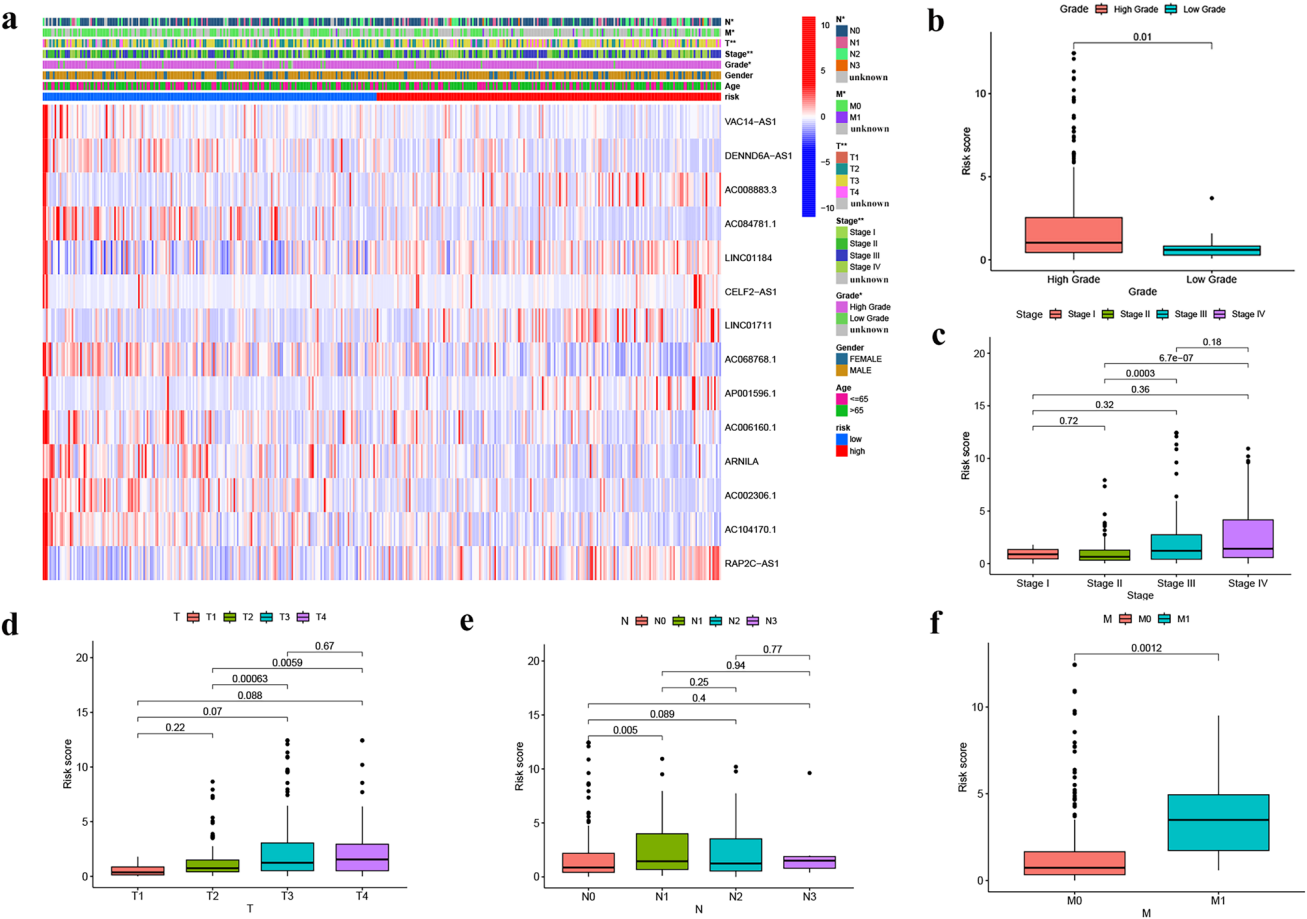
The telomerase related lncRNAs based on risk model was associated with the clinicopathological characteristics of patients with BLCA in the TCGA database. (**a**) Clinicopathological characteristics and heat map of 14 TERLs expression between the two risk groups. (**b**) Boxplot of risk score based on TERLs signature in BLCA patients with different pathological grades. (**c**) Boxplot of risk score based on TERLs signature in BLCA patients with different tumor stages. (**d**–**f**) Boxplot of risk score based on TERLs signature in BLCA patients with different T status, N statues and M status (*P < 0.05, **P < 0.01).

**Figure 4:**
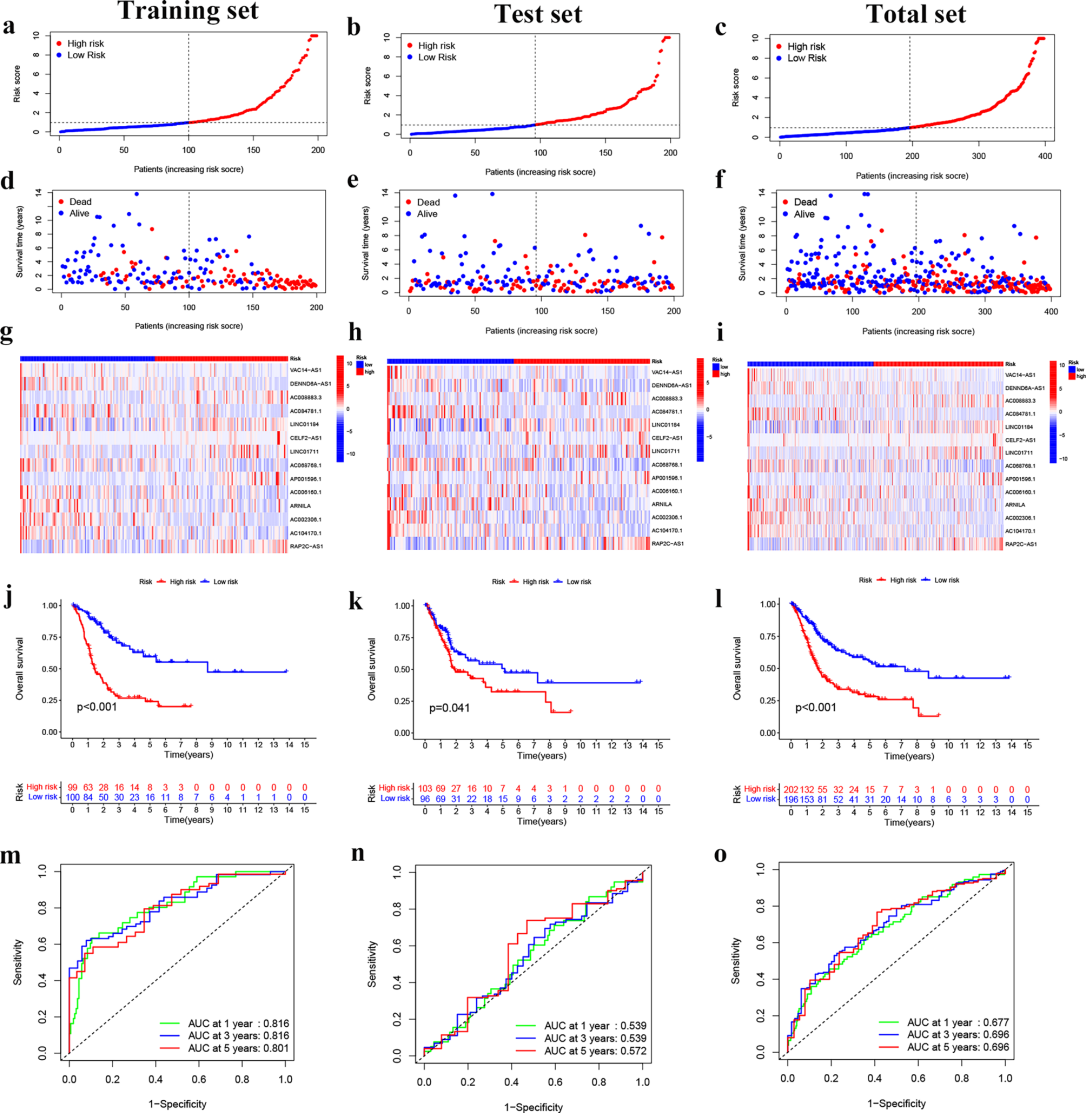
Risk model prognosis in different cohorts. (**a**–**c**) Risk curves on the basis of training, test, and total sets’ risk scores. (**d**–**f**) Survival state graphs according to training, test, and total sets’ risk scores. (**g**–**i**) Heatmap of risk cohorts and 14 TERLs in the training, test, and total sets. (**j**–**l**) Kaplan–Meier survival curves of overall survival of patients with BLCA. (**m**–**o**) The AUCs at 1-, 3-, and 5-years of prognostic models in the training, test and total sets, respective.

**Figure 5:**
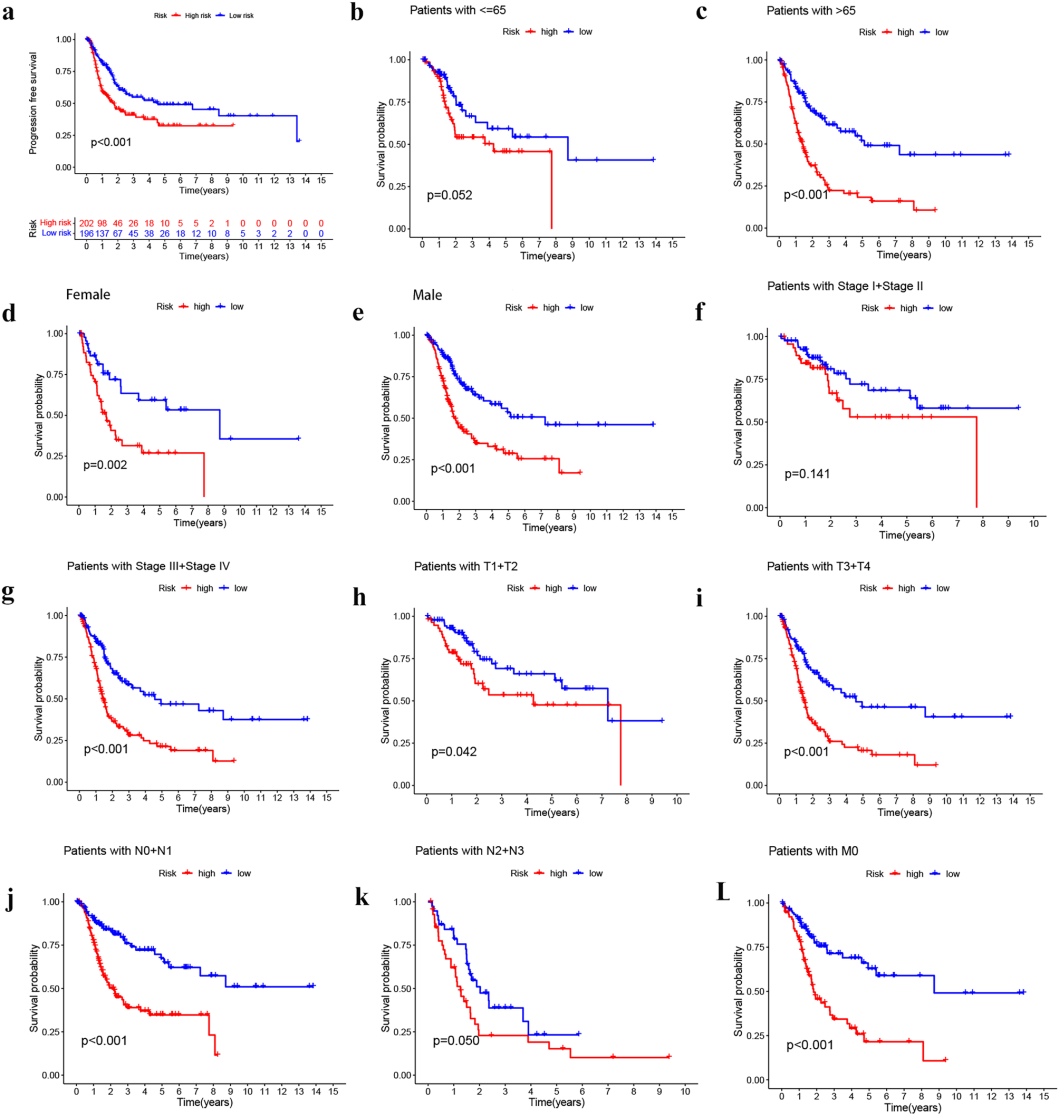
Kaplan–Meier survival curve for H-R and L-R cohorts by different clinical characteristics. (**a**) Progression free survival. (**b**, **c**) Age. (**d**, **e**) Sex. (**f**, **g**) Stage. (**h**, **i**) T stage. (**j**, **k**) N stage. (**l**) M stage.

In the TCGA-BLCA cohort, risk scores were correlated with clinical characteristics and presented as a heat map (Fig. 3a). Additional correlation analysis showed a statistically significant difference (P < 0.05) between risk score and pathologic grade (high grade vs low grade) (Fig. 3b), stage (stage II vs stage III, stage II vs stage IV) (Fig. 3c), T status (T2 vs T3, T2 vs T4) (Fig. 3d), N status (N0 vs N1) (Fig. 3e), and M status (M0 vs M1) (Fig. 3f). Table 1 shows that the clinical variables do not differ significantly between the training, test, and total sets. This indicates that there is no bias in clinical characteristics between the different sets. Then, BLCA patients were grouped into H-R and L-R cohorts based on median risk score. In the training, test, and total sets, the H-R cohort had a higher risk score and a higher mortality than the L-R cohort (Fig. 4a–i). The OS and progression-free survival (PFS) were substantially shorter in the H-R cohort (*P* < 0.05) (Figs. 4j–l and 5a). The AUC of 1-, 3- and 5-years of training set were 0.816, 0.816 and 0.801, respectively (Fig. 4m). For the test set, the AUC of 1-, 3- and 5-years were 0.539, 0.539 and 0.572, respectively (Fig. 4n). For the total set, the AUC of 1-, 3- and 5-years were 0.677, 0.696 and 0.696, respectively (Fig. 4o). When the correlation of OS with the risk score of TCGA-BLCA patients was investigated by clinical characteristics, the OS was markedly greater in the L-R cohort (Fig. 5b–e,g–j,l), except for stage N2 + N3 and stage I + stage II (Fig. 5f,k).

**Table 1:** Clinical characteristics of the total, test, and training sets.

Characteristics	Type	Total	Test	Training	P value
Age	≤ 65	158 (39.8%)	77 (38.89%)	81 (40.7%)	0.79
	> 65	239 (60.2%)	121 (61.11%)	118 (59.3%)	
Sex	Female	101 (25.44%)	45 (22.73%)	56 (28.14%)	0.26
	Male	296 (74.56%)	153 (77.27%)	143 (71.86%)	
Grade	High grade	374 (94.21%)	184 (92.93%)	190 (95.48%)	0.78
	Low grade	20 (5.04%)	11 (5.56%)	9 (4.52%)	
	Unknown	3 (0.76%)	3 (1.52%)	0 (0%)	
Stage	Stage I	2 (0.5%)	0 (0%)	2 (1.01%)	0.22
	Stage II	126 (31.74%)	66 (33.33%)	60 (30.15%)	
	Stage III	137 (34.51%)	73 (36.87%)	64 (32.16%)	
	Stage IV	130 (32.75%)	58 (29.29%)	72 (36.18%)	
	Unknown	2 (0.5%)	1 (0.51%)	1 (0.5%)	
T	T0	1 (0.25%)	0 (0%)	1 (0.5%)	0.19
	T1	3 (0.76%)	0 (0%)	3 (1.51%)	
	T2	116 (29.22%)	59 (29.8%)	57 (28.64%)	
	T3	189 (47.61%)	100 (50.51%)	89 (44.72%)	
	T4	56 (14.11%)	23 (11.62%)	33 (16.58%)	
	TX	1 (0.25%)	0 (0%)	1 (0.5%)	
	Unknown	31 (7.81%)	16 (8.08%)	15 (7.54%)	
M	M0	192 (48.36%)	92 (46.46%)	100 (50.25%)	1
	M1	11 (2.77%)	5 (2.53%)	6 (3.02%)	
	Unknown	194 (48.87%)	101 (51.01%)	93 (46.73%)	
N	N0	231 (58.19%)	124 (62.63%)	107 (53.77%)	0.16
	N1	44 (11.08%)	16 (8.08%)	28 (14.07%)	
	N2	75 (18.89%)	38 (19.19%)	37 (18.59%)	
	N3	6 (1.51%)	2 (1.01%)	4 (2.01%)	
	Unknown	41 (10.33%)	18 (9.09%)	23 (11.56%)

### Validation of signature and construction of the nomogram

**Figure 6:**
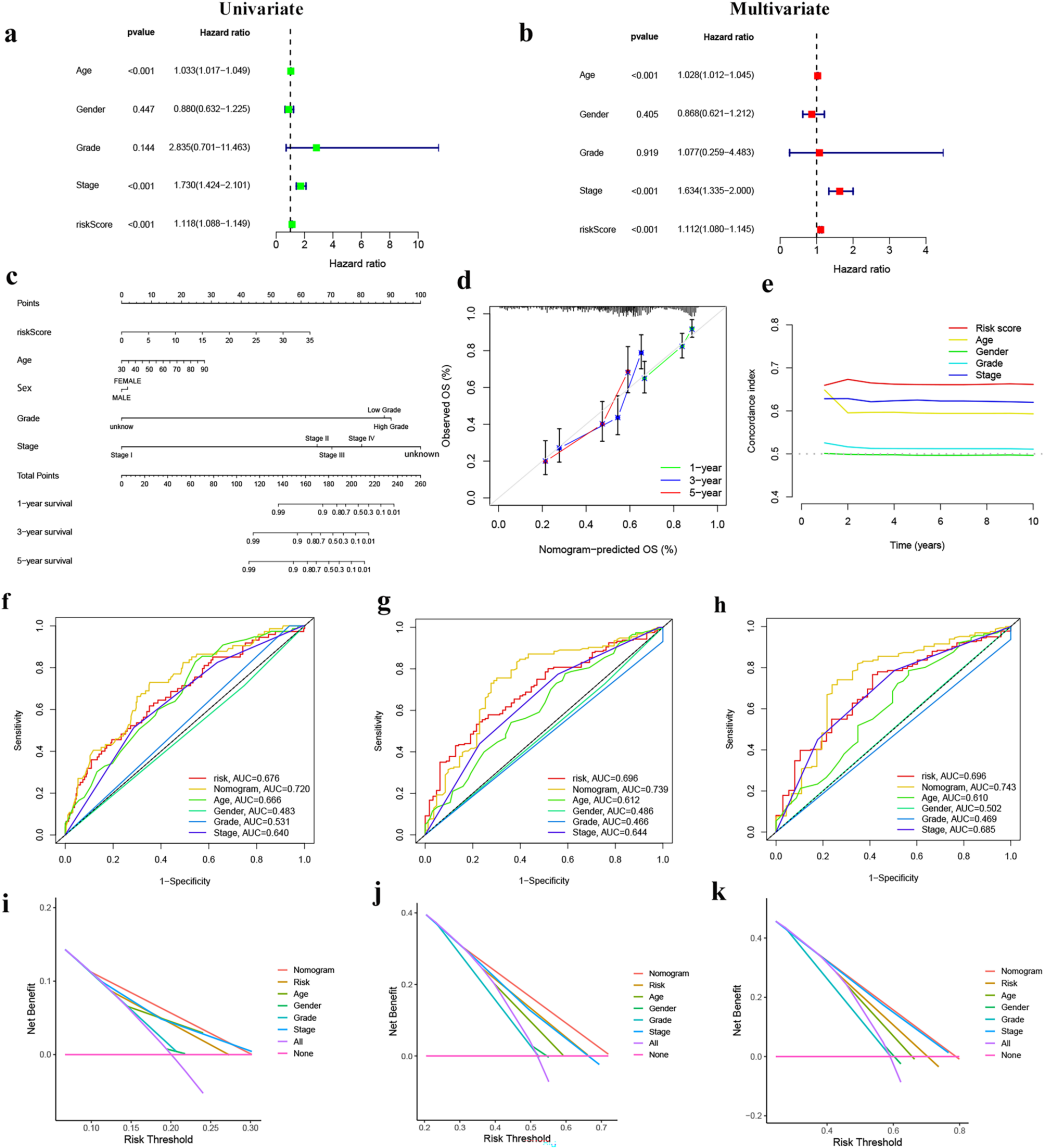
Construction and assessment of the prognostic model. (**a**) Univariate Cox regression analyses of clinical features and risk scores. (**b**) Multivariate Cox regression analyses of clinical features and risk scores. (**c**) The nomogram with integrated clinical characteristics and risk scores to predict the 1-, 3-, and 5-year OS. (**d**, **e**) The calibration curves and C-index of the clinical features and risk scores. (**f**–**h**) The AUCs of the nomograms compared for 1-, 3-, and 5-year OS, respective. (**i**–**k**) The DCA curves of the nomograms compared for 1-, 3-, and 5-year OS, respective.

Univariate and multivariate Cox regression assessments of risk score and clinical features for TCGA-BLCA confirmed that age (1.028, 95% CI 1.012–1.045, *P* < 0.001), stage (1.634, 95% CI 1.335–2.000, *P* < 0.001), and risk score (1.112, 95% CI 1.080–1.145, *P* < 0.001) were independent OS variables (Fig. 6a,b). Next, we employed a nomogram that incorporates both clinical features and a risk score to predict the prognosis of individuals with bladder cancer for 1-, 3-, and 5-year periods. To assess the accuracy of the nomogram, we plotted calibration curves. (Fig. 6c,d). The C-index revealed that the risk score of the model was superior to other clinical features (Fig. 6e). Figure 6f–h showed that the nomograms are more predictive of 1-, 3-, and 5-year survival that AUCs. DCA demonstrated that the nomogram provided the best clinical benefit (Fig. 6i–k), indicating a superior diagnostic value of the prognostic model.

### Data from PCA, functional enrichment analyses

**Figure 7:**
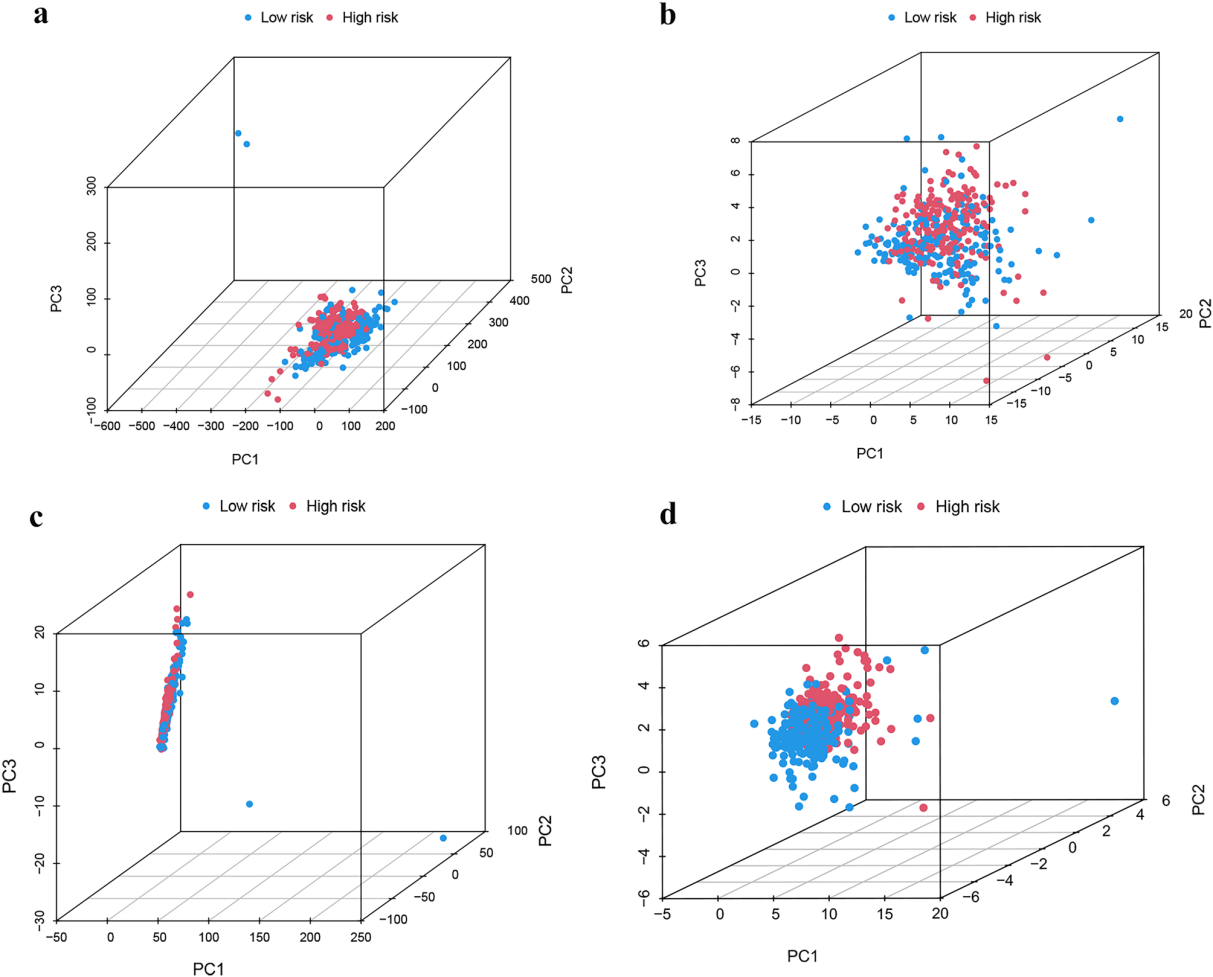
Principal component analysis between L-R and H-R cohorts. (**a**) All genes. (**b**) Telomerase genes, (**c**) Telomerase-related lncRNAs. (**d**) 14 Telomerase-related lncRNAs of the risk model.

**Figure 8:**
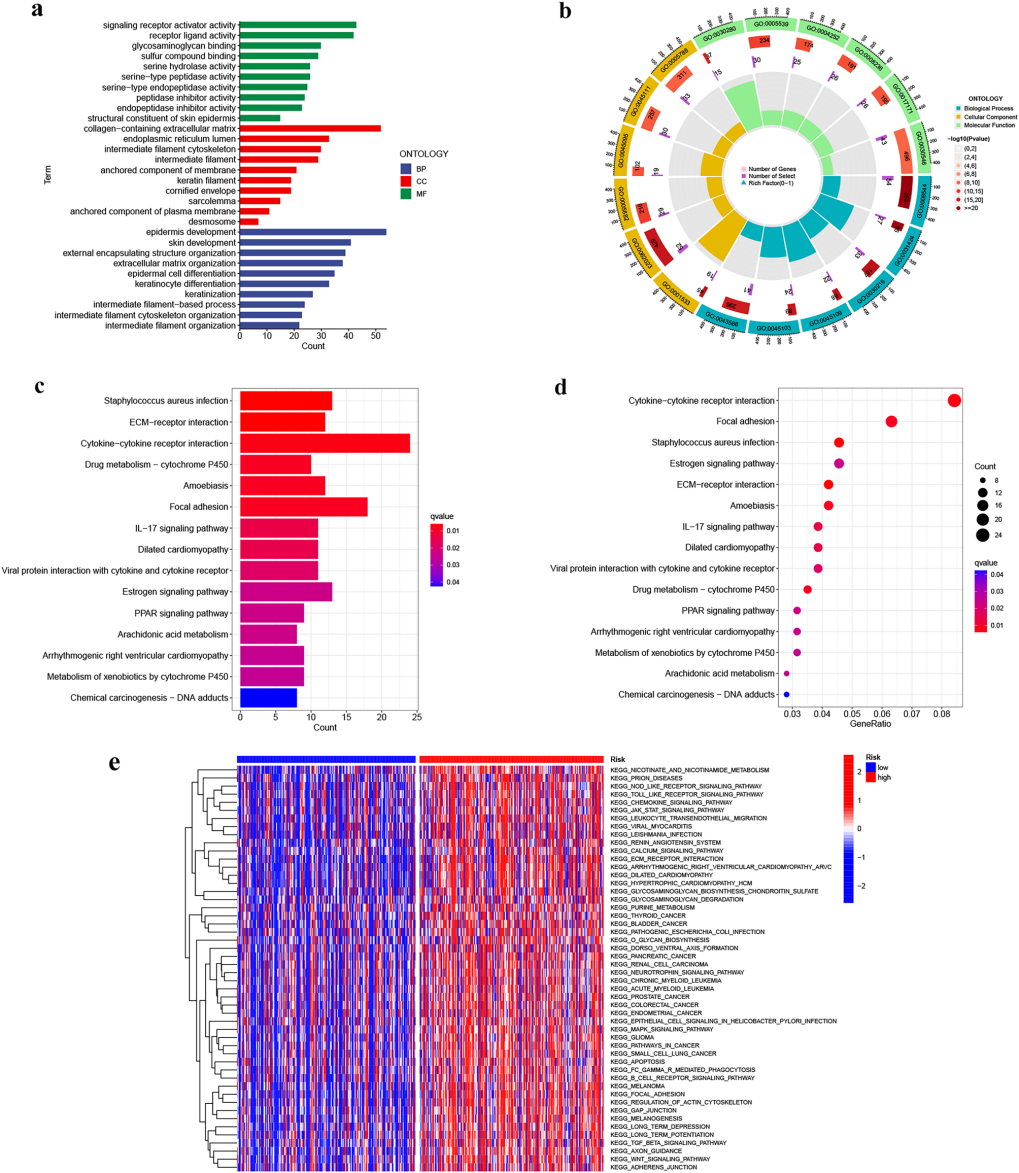
Functional enrichment analysis. (**a**, **b**) Important GO functional enrichment analysis terms. (**c**, **d**) Significant KEGG functional enrichment pathways. (**e**) The GSVA analysis between the H-R and L-R cohorts.

**Figure 9:**
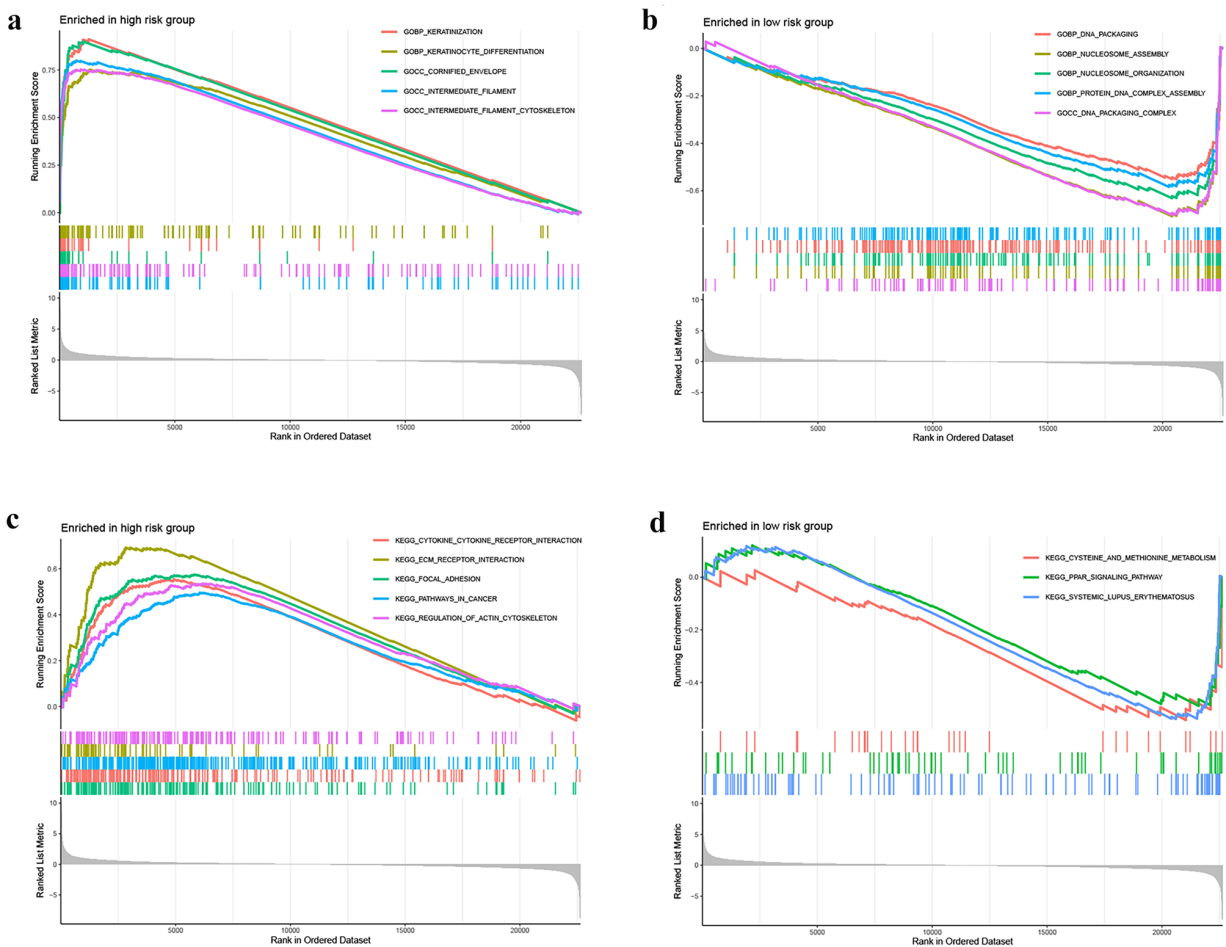
Enrichment plots from gene set enrichment analysis in the H-R and L-R cohorts according to risk score based on the risk model. (**a**, **b**) Significant enrichment in the two risk cohorts based based on GO functional annotation. (**c**, **d**) Significant enrichment in the two risk cohorts based based on KEGG functional annotation.

PCA analyses differentiated the four expression profiles (all gene expression, telomerase regulation genes, TERLs, and TERLs of the prognostic model) and indicated that the model could significantly differentiate the risk classification of BLCA patients (Fig. 7a–d). GO analysis revealed that TERLs were markedly elevated in signaling receptor activator activity, extracellular matrix comprising collagen, and epidermis development in BP, CC, and MF, respectively (Fig. 8a,b and Table S2). According to KEGG pathway analysis, TERLs were primarily increased in cytokine–cytokine receptor interaction, focal adhesion, and staphylococcus aureus infection (Fig. 8c,d ^26–28^ and Table S3). As for GSVA, top 50 highly enriched pathways were illustrated in Fig. 8e and Table S4. We further used GSEA for complementary and validated KEGG and GO functional annotations (Fig. 9a–d), detailed were showed in Tables S5 and S6.

### Tumor immune characteristics analysis

**Figure 10:**
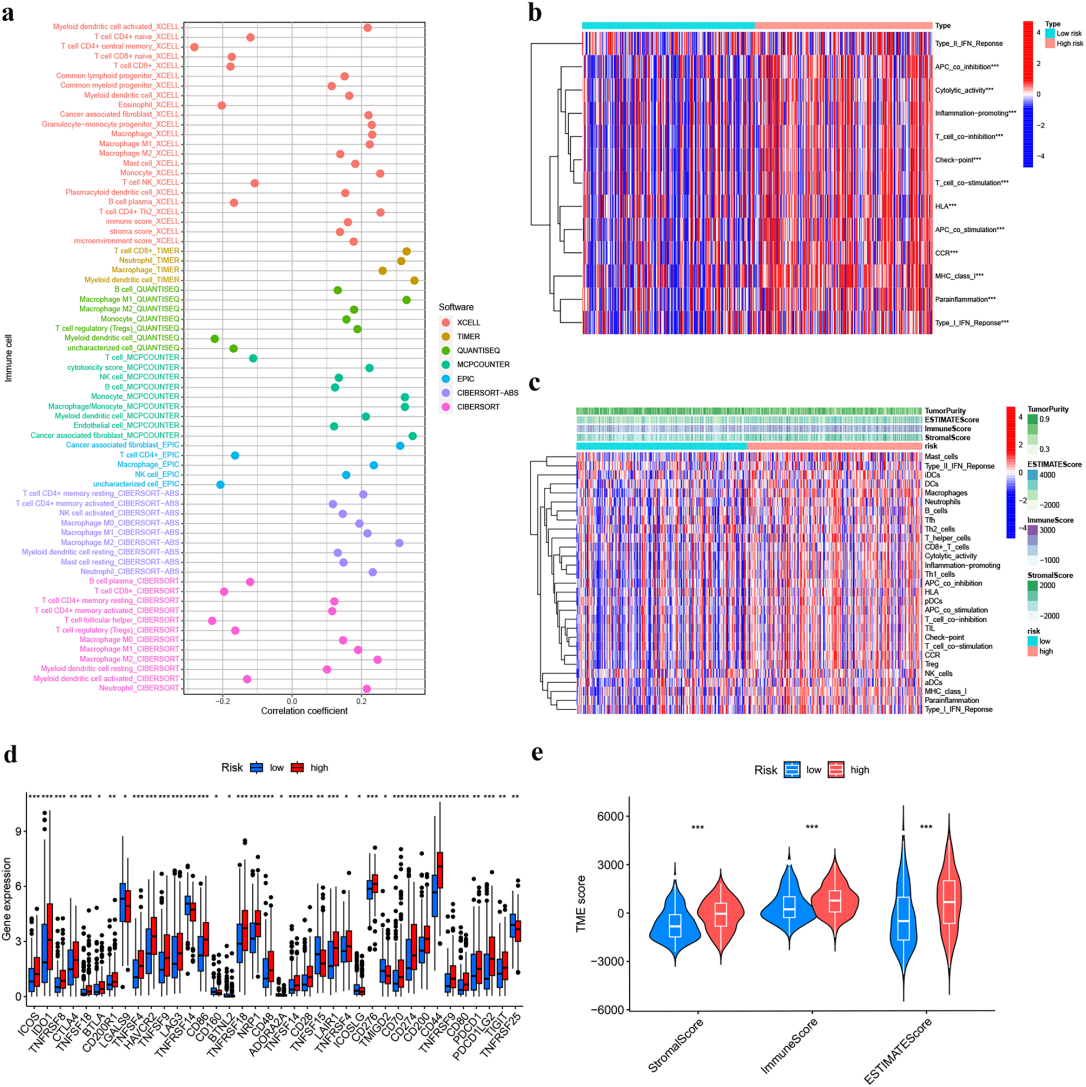
TME differences in H-R and L-R cohorts. (**a**) Immune cell bubble of different risk cohorts. (**b**) Comparison of immune pathways between the H-R and L-R cohorts. (**c**) Differences in immune cell infiltration in H-R and L-R cohorts. (**d**) Differences in expression of common immune checkpoints in the risk cohorts. (**e**) Comparison of Stromal Score, Immune Score, and ESTIMATE Score between L-R and H-R cohorts. (*P < 0.05, **P < 0.01, and ***P < 0.001).

Different algorithms analyzed the relationship of risk score and immune cell infiltration. It was concluded that many immune cells aggregated in the H-R cohort, including myeloid dendritic cells, T cell CD8+, and neutrophil in TIMER, cancer-linked fibroblast and macrophage in MCPCOUNTER, T cell CD4+ Th2 and Monocyte in XCELL (all *P* < 0.05) (Fig. 10a). Infiltrating immune cells' details were presented in Table S7. The results of ssGSEA indicated that the immune pathways' (APC_co_inhibition, T_cell_co_inhibitin, Check_point, T_cell_co_stimulation, HLA, APC_co_stimulation, etc.) activation status differed significantly between the two-risk cohorts (Fig. 10b). The TME score showed that the stromal and immune cells and ESTIMATE scores were elevated in the H-R compared to the L-R cohort, with a statistically marked difference (Fig. 10e) (*P < 0.05, **P < 0.01, and ***P < 0.001). Combining the TME and risk scores, the immune cell infiltration and immune pathways between the two risk cohorts were compared, and we further confirmed that the H-R cohort had higher immune cell infiltration and immune pathway activation than L-R cohort (Fig. 10c). Additionally, the immune checkpoint genes (CD274, CTL4, LAG3, PDCD1, and TIGIT) were higher in the H-R cohort (Fig. 10d). This may also explain why patients in the high-risk group have a worse prognosis, as immune checkpoint genes, such as CD274, are associated with tumor cells that evade immune surveillance^29^.

### Mutation and drug sensitivity assessment

**Figure 11:**
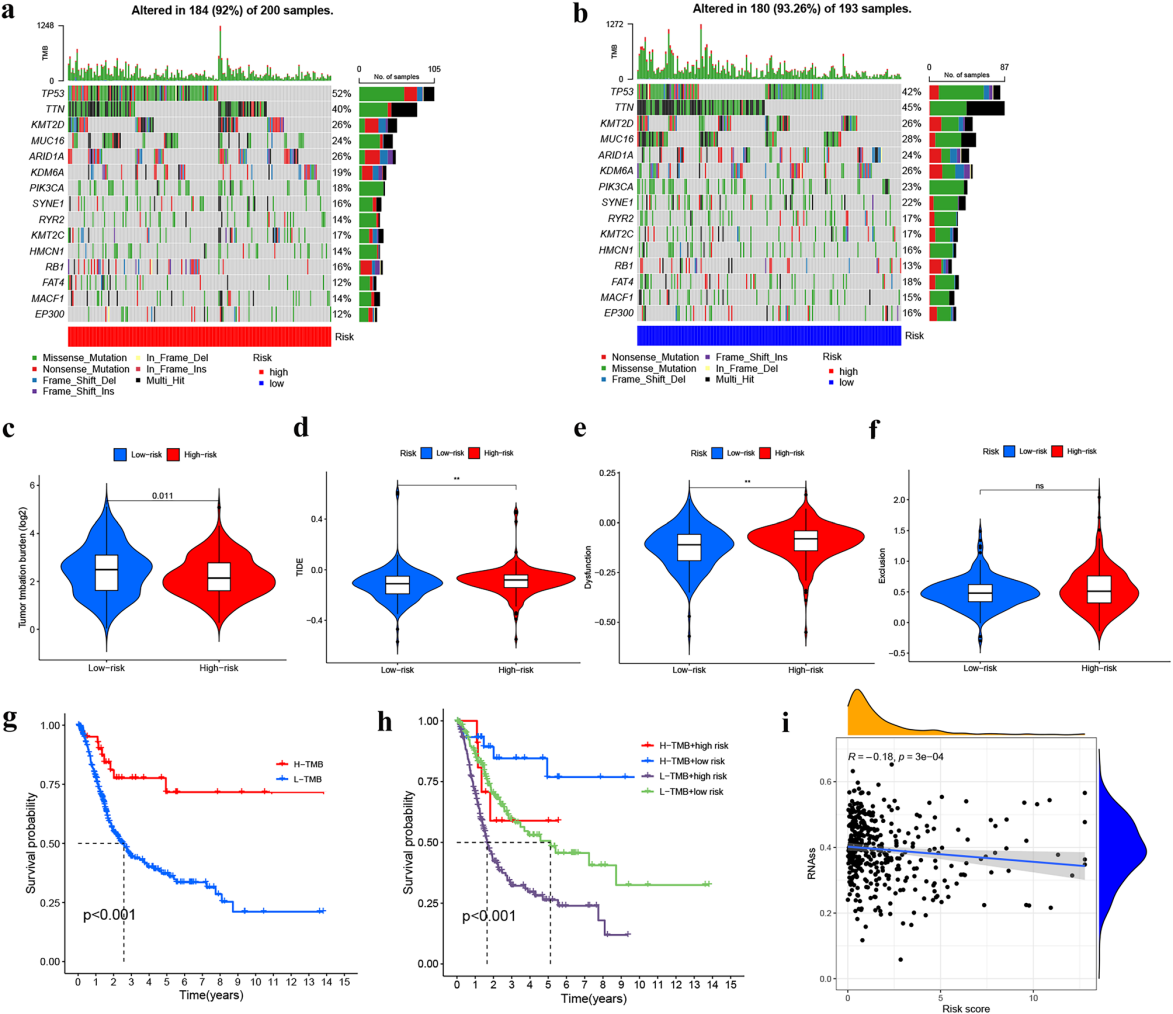
Somatic mutation, TMB, TIDE, and CSC scores in the risk cohorts. (**a**, **b**) Waterfall plots of somatic mutation characteristics in the two cohorts. (**c**) TMB between the H-R and L-R cohorts. (**d**–**f**) TIDE scores, Dysfunction scores, and Exclusion between the two cohorts. (**g**) Kaplan–Meier analysis of survival between the H-R and low-TMB cohorts. (**h**) Kaplan–Meier analysis of survival among 4 groups stratified by both TMB and risk cohorts. (**i**) The link between risk cohorts and the CSC index. (*P < 0.05, **P < 0.01, and ***P < 0.001).

**Figure 12:**
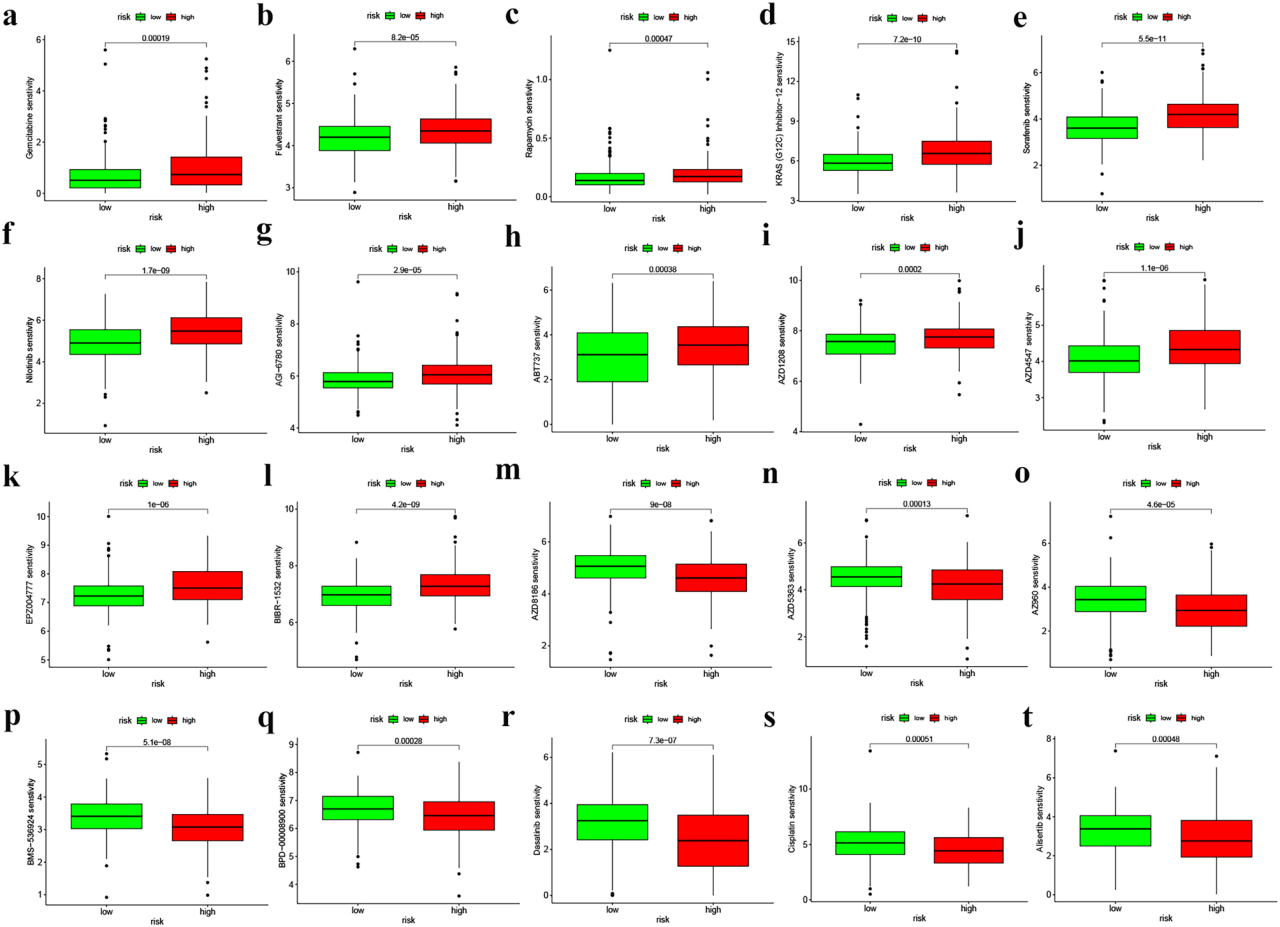
Prediction of drugs between the two risk cohorts. (**a**–**l**) Drugs are more sensitive in the L-R cohort. (**m**–**t**) Drugs more sensitive in a H-R cohort.

After analyzing the mutation data obtained from the TCGA-BLCA database using the Perl language, we identified the top 15 genes with the highest mutation frequencies in both risk cohorts. These genes are TP53, KMT2D, TTN, ARID1A, MUC16, KDM6A, SYNE1, PIK3CA, RYR2, HMCN1, KMT2C, RB1, MACF1, FAT4, and EP300 (as shown in Fig. 11a,b). Additionally, the TMB data obtained from the mutation data using Perl revealed that the L-R cohort had significantly higher TMB levels (P = 0.011) compared to other risk cohorts (Fig. 11c). The OS was notably increased in the high TMB cohort, suggesting that the H-R cohort with low TMB had the worst prognosis (Fig. 11g,h). TIDE score (TIDE score, dysfunction score, and exclusion score) is positively correlated with tumor immune escape potential. Our study found TIDE (Fig. 11d) and dysfunction scores (Fig. 11e) of the H-R cohort were higher compared to the L-R cohort (*P < 0.05, **P < 0.01, and ***P < 0.001), and no statistically significant difference was found in exclusion score (Fig. 11f). This suggests that the prognostic model has a high level of confidence in determining the effectiveness of immunotherapy. The CSC score was markedly negatively correlated (R = − 0.18, *P* < 0.001), revealing that BLCA stem cell characteristics and differentiation levels with lower risk scores are more prominent (Fig. 11i). Lastly, the risk model indicated that multiple drugs, such as gemcitabine, cisplatin, rapamycin, fulvestrant, sorafenib, etc., had significantly different IC50 in the two risk cohorts (Fig. 12a–t). All the drugs are displayed in Additional File 2 (Figs. S1 and S2).

### External validation of telomerase-related lncRNAs as a potential biomarker

**Figure 13:**
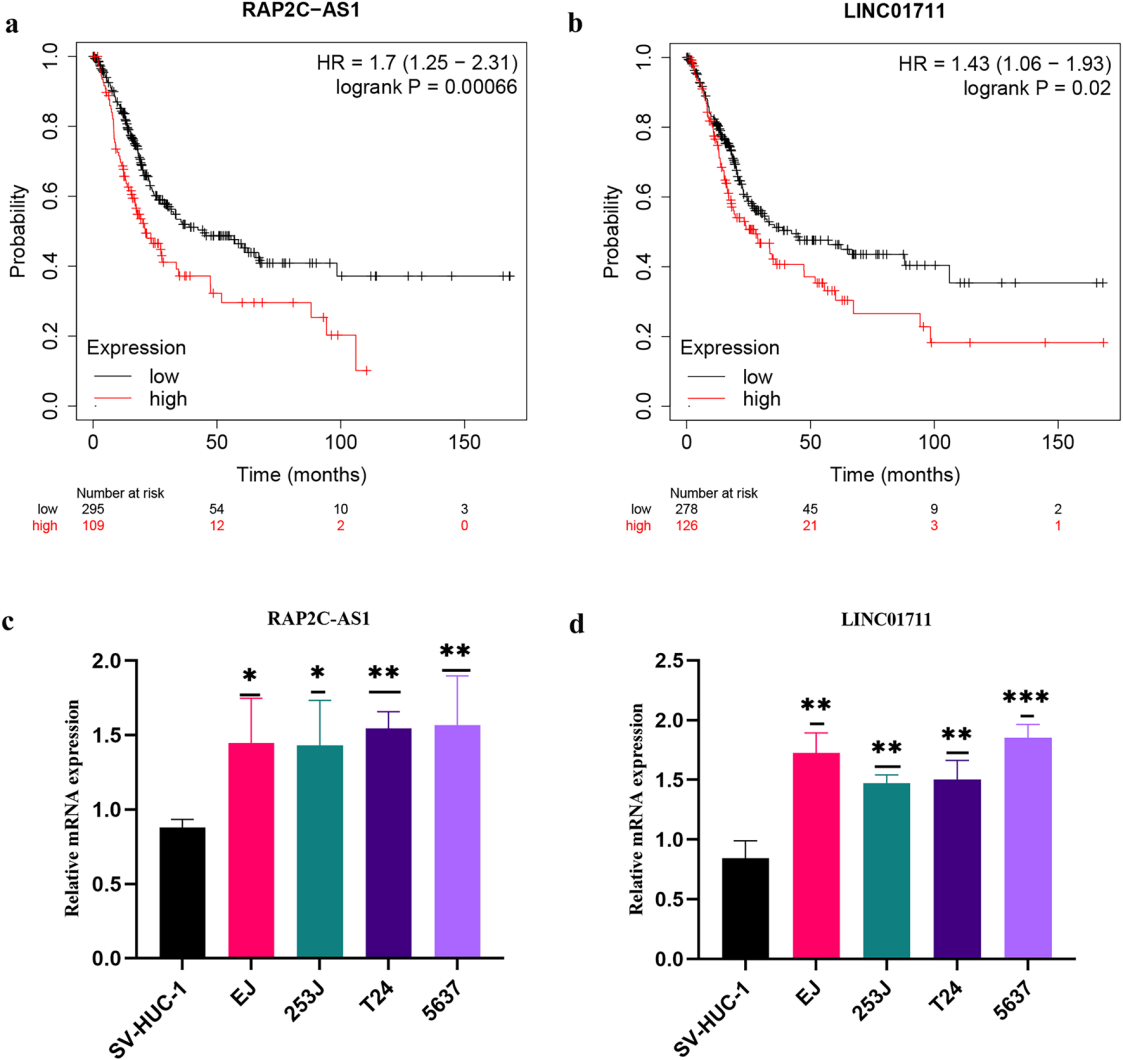
Validation of Telomerase-related lncRNAs as potential biomarkers. (**a**, **b**) OS analysis of RAP2C-AS1 and LINC01711 in the Kaplan–Meier Plotter Datasets. (**c**, **d**) the expression of RAP2C-AS1 and LINC01711in bladder cancer cell lines were measured by qRT-PCR (*P < 0.05, **P < 0.01, and ***P < 0.001).

**Figure 14:**
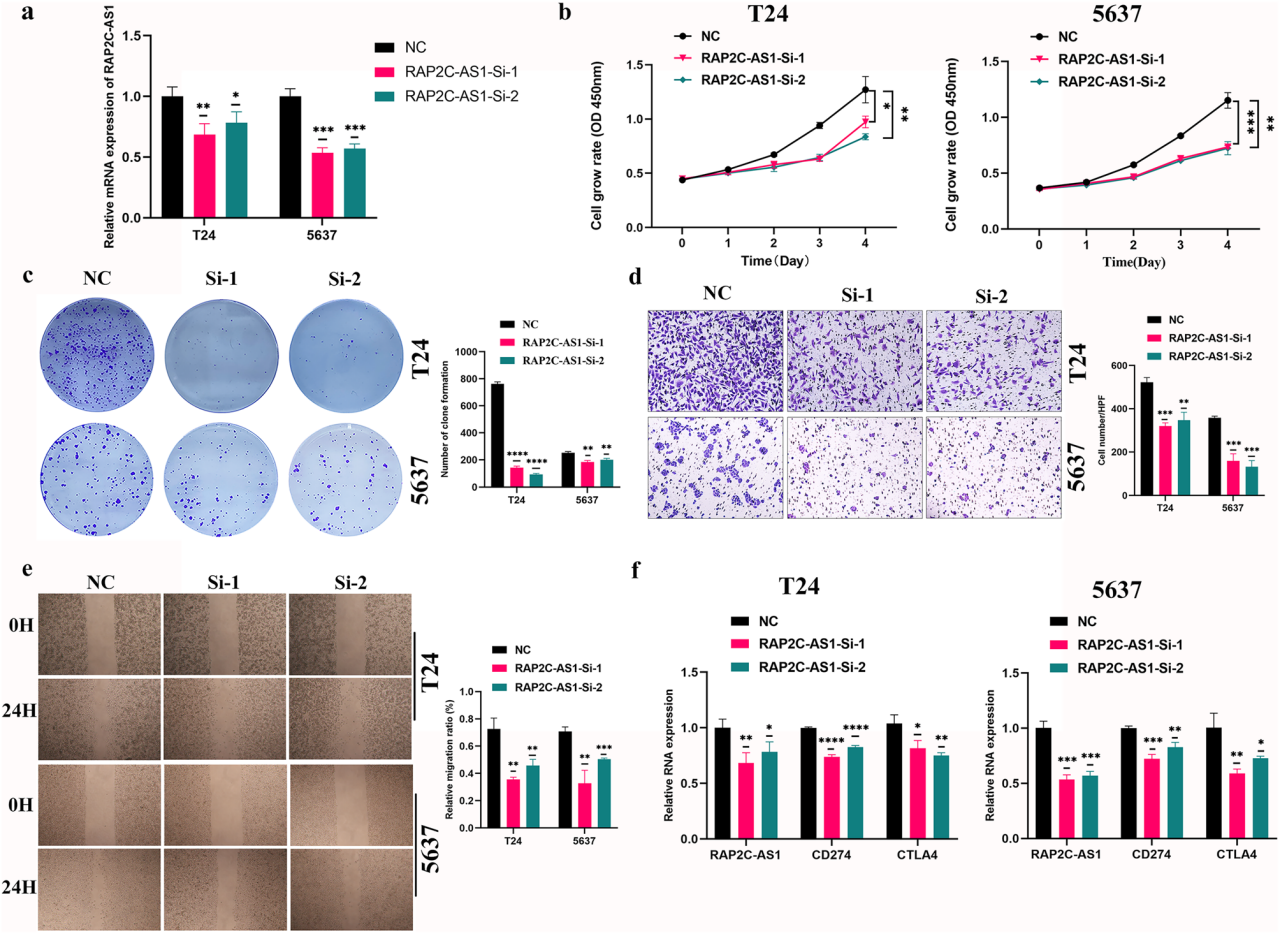
Effects of silencing RAP2C-AS1 expression on BLCA cells. (**a**) The mRNA knockdown efficiency of RAP2C-AS1 in T24 and 5637. (**b**) Effect of RAP2C-AS1 knockdown on the proliferation of BLCA cells showed by the CCK-8 assay. (**c**) Effect of RAP2C-AS1 knockdown on the proliferation of BLCA cells showed by the colony formation assay. (**d**) Effect of RAP2C-AS1 knockdown on the migration of BLCA cells showed by the transwell assays. (**e**) Effect of RAP2C-AS1 knockdown on the proliferation of BLCA cells showed by the wound healing assay. (**f**) Effect of RAP2C-AS1 knockdown on the mRNA expression of CD274 and CTLA4 (*P < 0.05, **P < 0.01, and ***P < 0.001).

Subsequently, we conducted a KM survival analysis to validate the prognostic potential of *RAP2C-AS1* and *LINC01711* using the Kaplan–Meier Plotter database. The findings demonstrated that *RAP2C-AS1* was significantly associated with OS (HR = 1.7 (1.25–2.31), logrank P = 0.00066) (Fig. 13a). Similarly, *LINC01711* was identified as a poor prognostic factor and correlated with OS (HR = 1.43 (1.06–1.93), log-rank P = 0.02) (Fig. 13b). The external dataset survival analysis results were consistent with our earlier findings. Additionally, we validated the expression of telomerase-related lncRNAs in BLCA cells (EJ, 253J, T24, and 5637) and SV-HUC-1 cells as the control group using qRT-PCR. The results indicated an overall trend of elevated expression of RAP2C-AS1 and LINC01711 in BLCA cells compared to that in SV-HUC-1 cells (Fig. 13c,d), which corresponded to our previous analysis based on the public database. RAP2C-AS1 was selected for additional in vitro validation. We selected T24 and 5637 for cell function experiments. The expression of RAP2C-AS1 was knocked down by siRNA, and the interference efficiency was verified at the RNA level (Fig. 14a). The results of CCK8 (Fig. 14b) and colony formation assays (Fig. 14c) showed that the knockdown expression of RAP2C-AS1 inhibited the proliferation of BLCA cells. In addition, the effect of RAP2C-AS1 knockdown on BLCA cell migration was investigated. Results from wound healing assay (Fig. 14e) and transwell assay (Fig. 14d) indicated that the migration ability of BLCA cells was significantly affected by the RAP2C-AS1 inhibition. Finally, to verify the role of RAP2C-AS1 in the tumor immune microenvironment, the relationship between RAP2C-AS1 and immune checkpoint genes (CD274 and CTLA4) was analyzed at the RNA level. The results indicated that the knockdown of RAP2C-AS1 led to a reduction in the expression of CD274 and CTLA4 (Fig. 14f), which were consistent with our previous analysis.

## Discussion

Telomere shortening is a process that is commonly associated with cell death, but it also plays a crucial role in maintaining stability in chromosomes. Telomerase elongates telomeres by re-expression of TERT catalytic subunits^30^. It is stimulated in stem cells and reduced in developing tissues. However, tumor cells are able to activate telomerase function and thus acquire the ability to self-replicate without restriction^31^. The mechanisms of TERT in cancer cells are diverse and include mutations and epigenetic regulation of promoters^32^. Approximately 60–80% of BLCA develops due to mutations in the promoter region. Kouchkovsky et al. reported that BLCA patients with TERT promoter mutations had higher exprssion of TMB and PD-L1 and were able to achieve better OS from ICIs^33^. Therefore, additional understanding of TERT and telomerase related research is urgently needed.

Numerous studies have found that lncRNA act as an essential regulator of tumorigenesis and BLCA development. Luo et al. confirmed that lncRNA RP11-89 competes endogenously to regulate ferroptosis and BLCA progression via the RP11-89/miR-129-5p/PROM2 axis and may modulate the immune microenvironment through PROM2^34^. Yang et al. reported that lncRNA ADAMTS9-AS1 promoted proliferation, migration, and reduced apoptosis and autophagy in 5637 and T24 cell lines through the PI3K/AKT/mTOR pathway^35^. However, the activity of TERLs in the BLCA is still undetermined. Therefore, in this survey, we constructed a model based on TERLs to predict BLCA individuals' prognosis, especially those with advanced stages.

In the present study, a prognostic model consisting of 14 TERL was constructed. Of these, LINC01184 plays a pivotal in the BLCA prognostic and immunotherapy models^36^. Wang et al. reviewed that LINC01184 is upregulated in hepatocellular carcinoma and could be used as its prognostic and diagnostic biomarker^37^. Sui et al. reported that LINC01184 may promote colorectal cancer proliferation and invasion via LINC01184-miR-331-HER2-p-Akt/ERK1/2 pathway by acting as a competing endogenous RNA^38^. LINC01711 is markedly expressed in esophageal cancer and has been linked to substandard prognosis by promoting tumor cells proliferation, migration, and invasion properties through FSCN1 upregulation and miR-326 downregulation^39^. Bakhya Shree et al. confirmed that LINC01711 also promotes glioblastoma multiforme cells' proliferating, migrating and invading properties and is associated with its substandard prognosis^40^. ARNILA has been reported to have poor PFS and stimulated EMT, invasion, and metastasis in triple-negative breast cancer patients in vitro and in vivo^41^. RAP2C-AS1 has been linked to prognostic model construction for clear cell renal cell carcinoma^42^. Liu et al. reported that AC006160.1 is a ferroptosis-related lncRNA; its overexpression significantly suppresses the proliferation and invasion of BLCA cell lines. In addition, patients with elevated AC006160.1 expression were sensitive to metformin and methotrexate^43^.

The remaining LncRNAs in the risk model were firstly reported in this investigation, providing new directions for BLCA research. Then, based on clinical characteristics and risk score, a nomogram was generated to predict the outcome of BLCA patients. GO analysis indicates that TERLs are significantly enriched in signaling receptor activator function, the extracellular matrix comprising collagen, and epidermis development. KEGG enrichment analysis revealed elevated lncRNAs in cytokine–cytokine receptor interaction nomogram and focal adhesion in the risk model. These pathways are believed to be associated with the prognosis and tumor immune microenvironment (TME) of gastric cancer and BLCA^44,45^, suggesting that TERLs were linked with TME and tumor development. The TME primarily comprises immune and stromal cells^46^. Analysis of the TME scores of BLCA patients in the two risk cohorts revealed that the immunization score, stromal score, and ESTIMATE score were higher in the H-R cohort. In addition, when the ssGSEA score for immune activity was compared between the two risk cohorts, the H-R cohort had elevated overall immunity and TME immunogenicity.

Theoretically, immune functional pathways with more immune cell infiltration and TME activation should have better immunotherapy outcome and prognosis^47^. However, in this investigation, BLCA prognosis in the H-R cohort was substantially poorer, probably due to the immune escape of tumor cells mainly because of the immunosuppressive microenvironment (immunosuppressive cells and cytokines). Tumor gene mutations have the potential to trigger the dysfunction of certain proteins or peptides, inducing the formation of new antigens that can be recognized by immune cells and initiating immune-mediated clearance of tumor cells with the ability to escape immune cell surveillance. Our research indicated that BLCA individuals in the H-R cohort had greater TIDE and dysfunction scores but lower TMB than the L-R cohort (P < 0.05). Recent studies have shown that TMB and TIDE are predictive biomarkers for immunotherapy^48^. When the expression of ICGs in both the risk cohorts was assessed, it was found that most of the ICGs (CD274, CTL4, LAG3, PDCD1, and TIGIT) were greatly expressed in the H-R cohort (all *P* < 0.05). This would also lead to a greater susceptibility of patients in the high-risk group to immunosuppression. Because immune checkpoint genes such as CD274 (PDL1) and CTLA4 can cause T-cell dysfunction, preventing cytotoxic T cells from targeting tumor cells and promoting tumor progression^49,50^. Gemcitabine, in combination with cisplatin, is the chemotherapy regimen of choice for BLCA, but resistance after chemotherapy is also becoming more common. The investigation has predicted 54 drugs that could be potentially effective for the two risk cohorts. It is believed that a precise treatment involving a rational combination of these drugs could reduce the incidence of drug resistance and improve the survival time for BLCA patients.

Despite the thorough bioinformatics, there were still many limitations in this investigation. First, the data were obtained from public databases, lacking validation from databases such as Gene Expression Omnibus (GEO) and International Cancer Genome Consortium (ICGC) databases. Efforts have been made to validate the potential of the screened TERLs as biomarkers using public databases, but no corresponding data sets have been found for their expression. Therefore, we conducted qRT-PCR experiments to validate the potential of two of these lncRNAs as biomarkers (RAP2C-AS1 and LINC01711), which were also verified using the external Kaplan–Meier Plotter database. RAP2C-AS1 was used for validation in vitro. Our results show that silencing RAP2C-AS1 significantly suppresses the proliferation and migration of BLCA cells. In addition, silencing RAP2C-AS1 down-regulates the expression of immune checkpoint genes such as CD274, reflecting its role in the tumor immune microenvironment. Moreover, the immune cell bubble plots present immunodeficiency results from multiple platforms and are considered external validation. Nonetheless, further validation using larger sample sizes and across multiple cancer types is necessary.

## Conclusion

In summary, we have screened 14 telomerase-related lncRNAs for prognostic signatures and developed a practical risk model to predict the OS in patients with BLCA. This model can be used to assess the prognosis and guide drug therapy, and the marker genes associated with telomerase may serve as current therapeutic targets for BLCA patients.

### Supplementary Information

Supplementary Figures.

Supplementary Table S1.

Supplementary Table S2.

Supplementary Table S3.

Supplementary Table S4.

Supplementary Table S5.

Supplementary Table S6.

Supplementary Table S7.

## Data Availability

The data of this study were obtained from the publicly available database. The RNA sequence and clinical information were acquired from TCGA database (https://portal.gdc.cancer.gov/ (accessed on 3, May, 2023). Tumor immune dysfunction and exclusion (TIDE) data from the TIDE database (http://tide.dfci.harvard.edu/ (accessed on 3, May, 2023). The telomerase gene set was acquired from the Gene set enrichment analysis (GSEA) website (http://www.gsea-msigdb.org/gsea/msigdb/ (accessed on 3, May, 2023). The bladder cancer cell lines involved in this article were obtained from the Institute of Urology, Second Hospital of Tianjin Medical University.
