# Supplementary figures and images for: Identification and validation of telomerase related lncRNAs signature to predict prognosis and tumor immunotherapy response in bladder cancer
Source: Sci Rep. 2023 Dec 9;13:21816. doi: 10.1038/s41598-023-49167-1 (PMC10710514; doi:10.1038/s41598-023-49167-1)

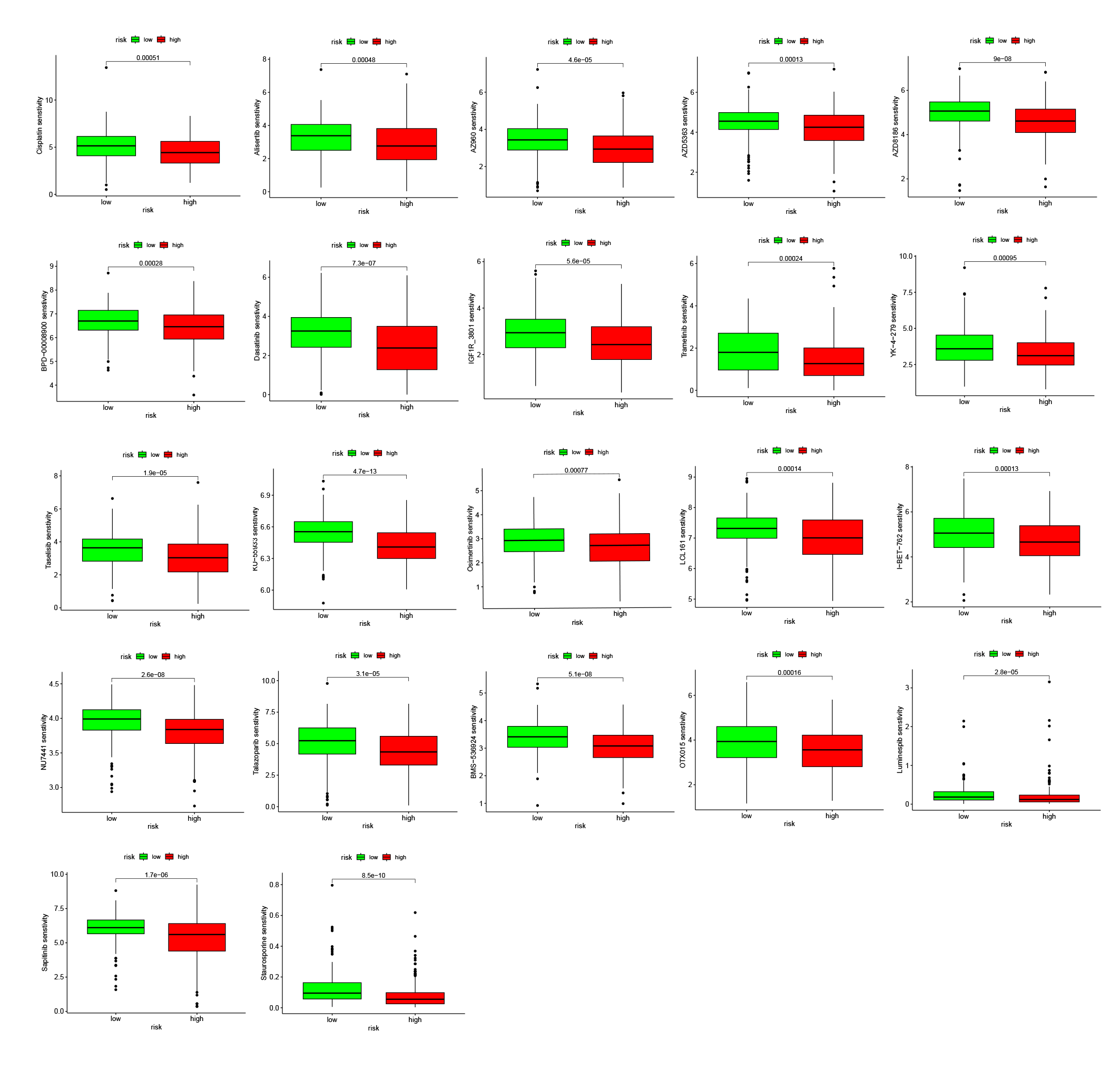


**Figure S1** Drugs more sensitive in high-risk group.

**
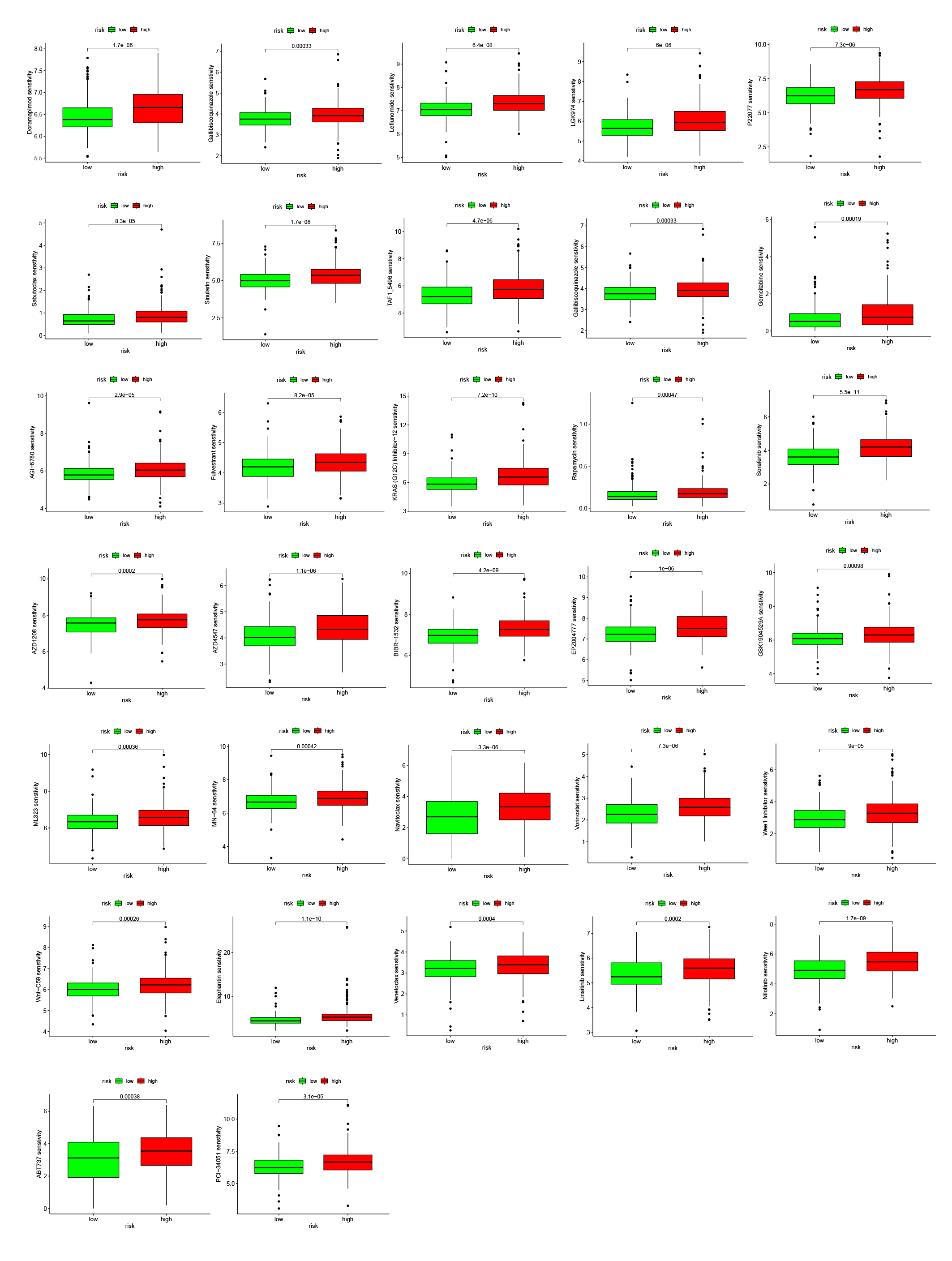
**

**Figure S2.** Drugs more sensitive in low-risk group.

Supplement: Supplementary file 1 — Supplementary Figures. [file 41598_2023_49167_MOESM1_ESM.docx]
